# Seasonal Difference in Antioxidant Capacity and Active Compounds Contents of *Eucommia ulmoides* Oliver Leaf

**DOI:** 10.3390/molecules18021857

**Published:** 2013-02-01

**Authors:** Qiang Zhang, Yinquan Su, Jingfang Zhang

**Affiliations:** College of Forestry, Northwest A&F University, 3 Taicheng Road, Yangling 712100, Shaanxi, China; E-Mails: zhangjack2003@yahoo.com.cn (Q.Z.); syq009@126.com (Y.S.)

**Keywords:** phenolics, flavonoids, *Eucommia ulmoides* Oliver, antioxidant, seasonal differences

## Abstract

Leaf of *Eucommia ulmoides* Oliver (EU) is a Traditional Chinese Medicine and a functional food in China. Antioxidant contents of EU leaves, which were collected monthly during the period of May–October in three years, were determined. Samples’ antioxidant capacity was characterized by DPPH radical scavenging activity, hydroxyl radical scavenging activity, ferrous chelating ability, and antioxidant capacity in linoleic acid emulsion and in rapeseed oil assays. The results showed that contents of some active compounds and antioxidant activity were related to a certain time of the year. Samples collected in August showed high content of phenolics, and the samples collected in May contained higher amount of flavonoids than other samples. Leaves collected in May or June exhibited high contents of rutin, quercetin, geniposidic acid and aucubin. The August leaves showed stable and high DPPH radical scavenging activity, and ferrous chelating ability. May samples showed strong inhibitory effects on oxidation of rapeseed oil and linoleic acid. The DPPH radical scavenging activity was related to the total phenolics content. Flavonoids played an important role in the inhibitory effects on rapeseed oil and linoleic acid oxidation. Therefore, August and May were indicated as the best months to harvest EU leaves for industry.

## 1. Introduction

*Eucommia ulmoides* Oliver (EU) is a unique medicinal plant grown in China. Cortex of EU has been used as a Traditional Chinese Medicine for thousands of years. In the past 20 years, the leaf of EU has become a popular functional health food and plant medicine material in China and Japan. EU tea (water extract of leaves) may reduce cholesterol and “fatty liver” [1], and also has been reported to reduce blood pressure [2]. It had a suppressing effect on mutagenicity and chromosome aberration following mutagen treatment [3,4], and it has shown a high antioxidant activity in recent studies, such as inhibition of oxidative damage in deoxyribose and DNA [5], and scavenging activity towards free radicals and reactive oxygen species (ROS) [6]. In addition, EU leaf showed a stronger antioxidant activity compared with the cortex, flower and fruit [7,8]. EU leaf is found to be rich of bioactive compounds such as polyphenolic acids (such as chlorogenic acid) and flavonoids (such as quercetin and rutin), iridoids (such as aucubin and geniposidic acid), as well as nutrients, *i.e.*, amino acids, vitamins and minerals [9,10,11,12]. Chlorogenic acid, a major bioactive compound found in the leaf, has demonstrated anti-lipid peroxidation, liver protection, antimicrobial and antiviral activity, and spasmolysis properties. Geniposidic acid is well known for its potential medicinal utilization, including prevention of sexual dysfunction, antioxidant activity, anti-aging, anti-inflammation, and anti-tumor [13]. Flavonoids also display many bioactive properties such as antiviral, anti-inflammatory, anti-allergic, and powerful anticancer functions [14]. Aucubin is beneficial for its bioactivities such as parasympathetic stimulation and anti-microbial activity [13].

For plant materials, the bioactive compounds content is different during its growing period. Thus, the bioactivity based on these constituents showed variability as well. That means it is important to study the seasonal differences in bioactivity and active compound contents. Unfortunately, although the EU leaf has been used as a functional food for over 20 years and for a traditional Chinese medicine for over 5 years, there is a little information about the seasonal variation of its bioactivity in a year. Some studies have reported different contents of flavonoids and phenolics of EU leaf, but all of the work was done within only one year [15,16,17,18,19], meaning that some random factors could affect the results. The aim of this work was to put forward a reference for industry to determine the optimum harvesting time for EU leaf. Hence, in our investigation, leaf was collected monthly from the same trees during the common sampling seasons in three different years. Their antioxidant activity and bioactive compound contents were determined and the results obtained compared with butylated hydroxytoluene (BHT), rutin, chlorogenic acid and ascorbic acid.

## 2. Results and Discussion

### 2.1. Phenolics and Flavonoids Contents

Phenolics are the most active antioxidants derived from plants [20]. The antioxidant activity of water extracts from EU leaves, bark and roasted bark had been correlated to their phenolics content (reported as gallic acid equivalents) [7]. The total phenolics compounds content (gallic acid equivalents) of all the extracts was determined using the Folin-Ciocalteu’s phenol reagent. As shown in Table 1, the highest total phenolics content in EU leaves is 1.20, 1.44, 1.46 times of the lowest value of leaves collected in 2009, 2010 and 2011, respectively. The data exhibited only small inter-monthly variations of total phenolic compounds content, but the samples collected in July and August still showed consistently higher phenolics contents than other seasons in the three year observations.

**Table 1:** Active compounds contents of EU leaves collected in different months (mg/g).

Harvest time	Flavonoids	Phenolic compounds	Rutin	Quercetin	Chlorogenic acid	Geniposidic acid	Aucubin
2009							
May	18.6 ± 2.31 ^a^	87.8 ± 3.91 ^c^	13.9 ± 2.02 ^a^	0.62 ± 0.06 ^a^	35.5 ± 1.17 ^c^	10.1 ± 1.19 ^c^	19.7 ± 0.28 ^a^
June	15.1 ± 1.46 ^b^	85.9 ± 4.40 ^c^	11.5 ± 2.44 ^b^	0.38 ± 0.03 ^b^	40.1 ± 2.84 ^b^	17.4 ± 2.31 ^a^	14.2 ± 2.10 ^b^
July	15.8 ± 2.63 ^b^	96.6 ± 7.65 ^b^	12.7 ± 0.84 ^b^	0.35 ± 0.01 ^b^	34.9 ± 2.54 ^c^	12.9 ± 1.77 ^b^	6.5 ± 0.85 ^d^
August	13.6 ± 1.73 ^b^	99.7 ± 2.90 ^b^	9.8 ± 1.75 ^c^	0.27 ± 0.03 ^c^	39.4 ± 1.98 ^b^	13.6 ± 0.39 ^b^	13.3 ± 1.46 ^b^
September	14.2 ± 1.37 ^b^	89.6 ± 6.29 ^c^	10.3 ± 1.53 ^c^	0.33 ± 0.04 ^b^	34.7 ± 2.65 ^c^	13.1 ± 2.42 ^b^	15.2 ± 1.97 ^b^
October	14.5 ± 3.94 ^b^	83.4 ± 5.24 ^c^	10.6 ± 0.97 ^c^	0.42 ± 0.02 ^b^	29.9 ± 3.08 ^d^	12.7 ± 1.05 ^b^	13.2 ± 2.32 ^b^
2010							
May	21.3 ± 2.74 ^a^	84.2 ± 6.65 ^c^	14.1 ± 1.55 ^a^	0.56 ± 0.06 ^a^	38.5 ± 3.96 ^b^	16.2 ± 0.69 ^a^	17.4 ± 2.47 ^a^
June	14.3 ± 2.11 ^b^	90.1 ± 1.42 ^c^	12.3 ± 1.87 ^b^	0.45 ± 0.07 ^b^	46.9 ± 1.06 ^a^	15.3 ± 2.15 ^a^	16.5 ± 1.39 ^a^
July	15.6 ± 1.06 ^b^	101.7 ± 5.97 ^b^	9.6 ± 1.11^c^	0.38 ± 0.05 ^b^	35.6 ± 1.19 ^c^	12.4 ± 1.73 ^b^	14.6 ± 0.48 ^b^
August	11.4 ± 0.97 ^c^	93.6 ± 2.61 ^b^	8.7 ± 0.98 ^c^	0.44 ± 0.08 ^b^	38.2 ± 3.21 ^b^	12.7 ± 0.69 ^b^	13.1 ± 1.96 ^b^
September	12.1 ± 1.63 ^c^	87.4 ± 8.39 ^c^	10.2 ± 0.75 ^c^	0.25 ± 0.03 ^c^	26.3 ± 1.25^d^	13.5 ± 1.63 ^b^	14.2 ± 1.52 ^b^
October	9.5 ± 1.72 ^c^	70.8 ± 6.61 ^d^	7.6 ± 2.15 ^d^	0.34 ± 0.03 ^b^	29.7 ± 4.01 ^d^	13.2 ± 2.04 ^b^	9.9 ± 0.34 ^c^
2011							
May	17.5 ± 0.88 ^a^	75.8 ± 9.43 ^d^	14.2 ± 0.84 ^a^	0.58 ± 0.05 ^a^	36.1 ± 2.35 ^c^	12.3 ± 1.29 ^b^	16.3 ± 1.05 ^a^
June	16.2 ± 1.79 ^b^	84.3 ± 6.49 ^c^	14.3 ± 1.48 ^a^	0.66 ± 0.08 ^a^	39.2 ± 1.86 ^b^	15.6 ± 0.93 ^a^	15.8 ± 1.93 ^a^
July	14.2 ± 1.28 ^b^	97.7 ± 4.32 ^b^	12 ± 1.87 ^b^	0.36 ± 0.03 ^b^	41.8 ± 2.21 ^b^	14.8 ± 1.08 ^a^	13.5 ± 2.36 ^b^
August	14.6 ± 2.06 ^b^	110 ± 5.78 ^a^	10.3 ± 1.59 ^c^	0.41 ± 0.08 ^b^	37.3 ± 2.38 ^c^	13.2 ± 1.86 ^b^	14.5 ± 1.23 ^b^
September	10.3 ± 1.83 ^c^	94.1 ± 9.59 ^b^	8.3 ± 0.68 ^d^	0.36 ± 0.05 ^b^	38.2 ± 3.45 ^b^	13.7 ± 1.59 ^b^	12.4 ± 0.48 ^b^
October	10.6 ± 0.41 ^c^	82.9 ± 6.76 ^c^	8.4 ± 1.17 ^d^	0.45 ± 0.04 ^b^	32.8 ± 3.08 ^d^	11.5 ± 1.31 ^b^	13.7 ± 1.34 ^b^

*Notes*: The values are the mean ± SD (n = 3). Significant differences at *p* < 0.05 are indicated with different letters within a column.

**Figure 1:**
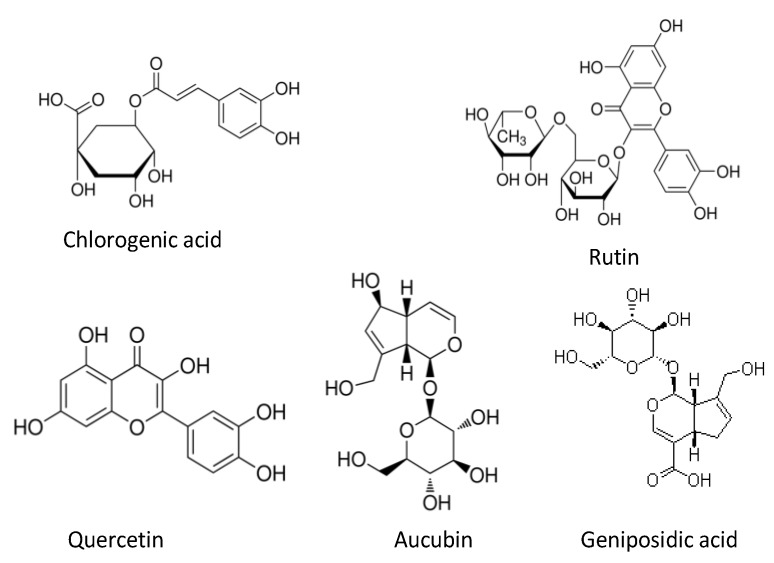
Structure of compounds in EU leaves.

Chlorogenic acid (structure shown in Figure 1) is an important phenolic compound in EU leaves. It has many kinds of bioactivities, including antioxidant activity [21]. As shown in Table 1, the highest content of chlorogenic acid in EU leaves is 1.34, 1.78, 1.27 times the lowest value of leaves collected in 2009, 2010 and 2011, respectively. The fluctuations of the chlorogenic contents were not too big. However, in the three years the samples collected in spring and summer consistently showed a slight superiority in chlorogenic acid content compared to leaves harvested in autumn.

Flavonoids are a type of natural compounds with strong activity and exist in many plants [22]. Expressed as rutin equivalents in milligrams per gram of dry leaf sample, the flavonoid contents in EU leaves are displayed in Table 1. As shown, the highest flavonoids content in EU leaves is 1.37, 2.24, 1.70 times the lowest content of leaves collected in 2009, 2010 and 2011, respectively. The inter-monthly variation of flavonoids content is similar to that of the total phenolics, but the highest content appeared in the samples of May in all of samples.

Rutin and quercetin (structure shown in Figure 1) are the main components in EU leaves flavonoids [18]. The highest rutin content in EU leaves is 1.42, 1.86, 1.72 times the lowest content of leaves collected in 2009, 2010 and 2011, respectively. The highest content of quercetin is 2.30, 2.24, 1.83 times the lowest value of 2009, 2010 and 2011, respectively. Like total flavonoids, the highest contents of rutin or quercetin were found in the samples of May or June, also during the warm season.

Geniposidic acid and aucubin (structure see in Figure 1) are important bioactive compounds in EU leaves and show relatively high contents [16]. As shown in Table 1, the highest content of geniposidic acid is 1.72, 1.31, 1.36 times the lowest content in 2009, 2010 and 2011, respectively. And the maximum content of aucubin is 3.03, 1.76 and 1.31 times of the minimum of 2009, 2010 and 2011, respectively. Like rutin and quercetin, the maximums of geniposidic acid and aucubin were exhibited in the leaves of May or June during the three years observed.

For the active compounds tested, May or August seems an appropriate time for harvesting. The aim of this research was to find the seasonal variation of samples, thus it is very important to prevent the interference from geological factors, soil factors and plant individual difference. After the same sampling from the same trees, examining the same analysis of EU leaves in the last three years, contents of flavonoids, total phenolics, chlorogenic acid, rutin, quercetin, geniposidic acid and aucubin continuously showed a peak related to a certain season.

### 2.2. Antioxidant Capacity

Food antioxidants are defined as “any substance that in small quantities is able to prevent or retard the oxidation of easily oxidizable materials such as fat” [23]. For evaluation of the antioxidant activity of EU leaf, the inhibition effects on the peroxidation of linoleic acid and rapeseed oil which is a popular food oil in China were investigated every day during the testing period. Determination of the inhibitory activity on rapeseed oil oxidation is one of the Chinese authorized methods of evaluation of activity of food antioxidants. As shown in Table 2, the highest inhibition activity against oxidation of linoleic acid is 3.12, 1.45, 2.25 times the lowest value for leaves collected in 2009, 2010 and 2011, respectively. The strongest inhibitory activity against oxidation of rapeseed oil is 1.66, 2.03, 1.30, times the lowest value of leaves collected in 2009, 2010 and 2011, respectively. As shown in Table 3, a statistically significant correlation between oil oxidation inhibition activities and flavonoids was observed. Correlation coefficients are 0.704 and 0.591 for rapeseed oil and linoleic acid, respectively. As the main components in EU flavonoids, rutin and quercetin showed high correlations with oil oxidation inhibition activities as well. The correlation coefficients are 0.674, 0.721 for rapeseed oil and 0.629, 0.480 for linoleic acid, respectively. Chlorogenic acid and aucubin also showed a correlation with inhibition of oxidation of oil, with correlation coefficients of 0.552 and 0.571, respectively.

**Table 2:** Antioxidant effects of EU leaves collected at different times.

Harvest time	Antioxidant activity (Rapeseed oil)	IC_50_ (DPPH)	Chelating ability (Fe^2+^)	Inhibition effect (linoleic acid)	Inhibition effect (OH^•^)
	(%)	(μg/mL)	(%)	(%)	(%)
2009					
May	63.00 ± 5.82 ^a^	147.1 ± 21.3 ^d^	68.94 ± 2.15 ^a^	67.27 ± 3.51 ^b^	35.93 ± 3.15 ^b^
June	58.17 ± 4.37 ^a^	135.3 ± 15.0 ^c^	70.11 ± 3.55 ^a^	58.82 ± 3.80 ^b^	15.93 ± 2.15 ^d^
July	47.51 ± 6.61 ^b^	152.9 ± 15.8 ^d^	68.73 ± 3.82 ^a^	64.43 ± 5.58 ^b^	24.84 ± 3.21 ^c^
August	42.89 ± 3.22 ^b^	134.4 ± 17.2 ^c^	72.11 ± 4.61 ^a^	45.26 ± 2.29 ^c^	41.54 ± 2.14 ^b^
September	43.54 ± 5.56 ^b^	189.7 ± 14.7 ^e^	28.63 ± 3.90 ^c^	32.28 ± 4.09 ^d^	37.91 ± 4.64 ^b^
October	43.96 ± 4.48 ^b^	215.2 ± 16.0 ^f^	60.67 ± 1.47 ^b^	34.36 ± 2.19 ^d^	17.03 ± 5.10 ^d^
2010					
May	68.35 ± 3.56 ^a^	162.5 ± 10.6 ^d^	60.76 ± 2.38 ^b^	66.47 ± 4.44 ^b^	44.18 ± 2.89 ^b^
June	50.72 ± 4.82 ^b^	148.8 ± 16.3 ^d^	47.95 ± 1.17 ^c^	62.85 ± 3.26 ^b^	43.63 ± 2.17 ^b^
July	49.84 ± 5.27 ^b^	135.3 ± 13.7 ^c^	57.68 ± 3.59 ^b^	49.22 ± 5.32 ^c^	19.39 ± 4.73 ^d^
August	46.73 ± 2.91 ^b^	136.7 ± 7.6 ^c^	75.32 ±3.25 ^a^	56.72 ± 2.85 ^b^	28.74 ± 5.21 ^c^
September	37.69 ± 4.59 ^c^	200.4 ± 15.8 ^e^	48.94 ± 3.31 ^c^	47.84 ± 6.17 ^c^	39.82 ± 5.34 ^b^
October	33.73 ± 2.14 ^c^	258.2 ± 21.5 ^g^	60.96 ± 1.81 ^b^	38.93 ± 2.37 ^d^	19.73 ± 5.26 ^d^
2011					
May	61.44 ± 6.35 ^a^	153.7 ± 16.6 ^d^	59.61 ± 3.64 ^b^	69.25 ± 6.37 ^b^	38.71 ± 5.73 ^b^
June	65.39 ± 3.28 ^a^	157.8 ± 9.4 ^d^	68.49 ± 2.88 ^a^	57.77 ± 4.92 ^b^	45.36 ± 4.98 ^b^
July	48.51 ± 5.44 ^b^	142.6 ± 17.5 ^c^	64.36 ± 2.81 ^b^	50.37 ± 3.63 ^c^	29.13 ± 2.11 ^c^
August	57.22 ± 2.79 ^a^	122.3 ± 12.6 ^c^	73.32 ± 1.59 ^a^	54.52 ± 3.53 ^c^	35.08 ± 6.27 ^b^
September	53.75 ± 3.53 ^b^	168.5 ± 11.4 ^d^	64.63 ± 2.05 ^b^	38.16 ± 4.72 ^d^	27.93 ± 4.18 ^c^
October	50.24 ± 5.75 ^b^	194.9 ± 13.5 ^e^	59.57 ± 2.72 ^b^	30.73 ± 2.65 ^e^	25.82 ± 4.52 ^c^
Compounds					
BHT	65.06 ± 2.35 ^a^	92.1 ± 14.8 ^b^	−0.22 ± 3.37 ^d^	84.93 ± 4.24 ^a^	96.08 ± 3.02 ^a^
Ascorbic acid	37.86 ± 3.74 ^c^	15.0 ± 5.5 ^a^	67.72 ± 3.42 ^a^	49.66 ± 5.11 ^c^	40.82 ± 4.75 ^b^
Rutin	84.41 ± 4.92 ^d^	21.2 ± 3.6 ^a^	79.37 ± 4.81 ^a^	88.35 ± 6.79 ^a^	64.39 ± 3.92 ^e^
Chlorogenic acid	63.84 ± 3.78 ^a^	118.4 ± 9.9 ^c^	36.55 ± 2.95 ^c^	82.77 ± 3.08 ^a^	19.84 ± 4.77 ^d^

*Notes*: The values are the mean ± SD (*n* = 3). Significant differences at *p* < 0.05 are indicated with different letters within a column. The concentration of samples was 1 mg/mL in the measurement of chelating effect, inhibition effect on hydroxyl radical (OH^•^) and inhibition effect on peroxidation of linoleic acid and rapeseed oil.

All of the plant samples inhibited the oxidation of rapeseed oil, but the activity was lower than that of BHT, rutin and chlorogenic acid. Samples collected in May showed stable and high activity among all plant samples in the three years tests, and their activity was close to that of BHT, exhibiting potential as a food antioxidant. From August, the inhibition effect of EU leaves on oil oxidation went down significantly.

In cells, free radicals are continuously produced either as by-products of metabolism or deliberately as in phagocytes [24]. The DPPH radical scavenging model is especially useful in evaluating chain-breaking activity in the propagation phase of lipid (and protein) oxidation [25]. The effect of antioxidants on DPPH radical scavenging was thought to be a result of their hydrogen donating ability [26]. The highest IC_50_ values of scavenging activities against DPPH radical were 1.60, 1.91, 1.59 times the lowest value of leaves collected in 2009, 2010 and 2011, respectively. From the three years of data in Table 3, DPPH scavenging activity showed a closer relation with total phenolics and chlorogenic acid, which is the main phenolic compound in EU leaves, than flavonoids; correlation coefficients are 0.694 and 0.703, respectively. That means phenolics play a more important role in DPPH radical scavenging activity in EU leaf than flavonoids, and chlorogenic acid is a main scavenger, although its activity is much lower than that of rutin. Leaves collected in August showed a stable and strong DPPH^•^ scavenging activity. In autumn, the activity decreased very fast. Nevertheless, none of the samples and BHT could be comparable with ascorbic acid and rutin whose IC_50_ values were 15.0 µg/mL and 21.2 µg/mL, respectively.

**Table 3:** Correlation coefficients of the compounds contents with the antioxidant activity.

Compounds	Antioxidant activity	IC_50_ (DPPH)	Chelating effect (Fe^2+^)	Inhibition effect (linoleic acid)	Inhibition effect (OH^•^)
	(rapeseed oil)				
flavonoids	0.704	−0.435	0.054	0.591	0.298
phenolics	0.144	−0.695	0.212	0.093	−0.072
rutin	0.674	−0.382	0.094	0.629	0.427
quercetin	0.721	−0.203	0.286	0.480	0.315
chlorogenic	0.552	−0.703	0.379	0.437	0.248
geniposidic acid	0.252	−0.179	0.091	0.209	0.131
aucubin	0.571	−0.287	−0.087	0.264	0.467

Hydroxyl radicals react with several biological materials oxidatively by removing hydrogens, double-bond addition, electron transfer and radical formation, and initiate autoxidation, polymerization and fragmentation processes. They are the most reactive oxygen species (ROS) [27]. As shown in Table 2, the highest inhibition activity against hydroxyl radical is 2.61, 2.28, 1.76 times the lowest value of leaves collected in 2009, 2010 and 2011, respectively. No common maximum or minimum in inhibition activity was related with a certain time of the year. As shown in Table 3, no statistical correlation was observed between hydroxyl radical inhibitory activity and flavonoids, total phenolics or other compounds determined, except for aucubin and rutin; correlation coefficients are 0.467 and 0.427, respectively, pointing out the possible important role of aucubin and rutin in this activity and the relatively high activity of rutin (64.39%) partly supported the speculation.

Metal ion chelating capacity is claimed as one of the antioxidant activity mechanisms [28,29], since it reduces the concentration of the catalyzing transition metal in lipid peroxidation [30]. Ferrozine can quantitatively form complexes with Fe^2+^. In the presence of EU leaves or added antioxidants the complex formation is disrupted, resulting in a decrease of the red color of the complex. As shown in Table 2, all of the plant samples and ascorbic acid exhibited a certain metal ion chelating ability. The highest metal ion chelating ability is 2.52, 1.54, 1.35 times the lowest value of leaves collected in 2009, 2010 and 2011, respectively. Three August samples showed the strongest ability in all of the three years measurements. Only chlorogenic acid showed a small statistical correlation (the correlation coefficient is 0.379) with the metal ion chelating ability, suggesting that many kinds of compounds in EU leaves could combine with the metal ion.

Although the factors involved in plant antioxidant activity are complex, and the data are more irregular than for the active compounds contents, some regular patterns were still repeated in the three years. The August leaves showed stable and high DPPH radical scavenging activity and metal ion chelating ability. May samples maintained strong inhibition effects on the oxidation of rapeseed oil and linoleic acid during the three years. EU leaves collected in September or October exhibited weak activity, therefore, May or August should be a good time for harvesting EU leaves.

Correlation analysis proved the connection between DPPH radical scavenging activity and total phenolics content and the important role of chlorogenic acid. Flavonoids and its main components, rutin and quercetin, were closely related to the inhibition effects on oil and linoleic acid oxidation. The high inhibition activity on oxidation of oil and linoleic acid of rutin and the relatively high DPPH scavenging activity and high content of chlorogenic acid may partly explain their important role in those assays. Flavonoids and phenolics include many types of compounds and these compounds show very different activities because of their various structures [22,31]. It is easier to observe the relationship between their contents and the antioxidant activity when the contents show significant differences. However, when the flavonoids or phenolics contents of the samples are close to each other, the property and content of every active compound need to be considered.

## 3. Experimental

### 3.1. Materials and Chemicals

EU leaves were collected on one day between the 18th and 25th of every month from May to October in 2009, 2010, and 2011 in a garden of Northwest A&F University, Yangling, China. The sampling place has a loess type soil, an elevation of 445.0 meters, annual average temperature of 13 °C, annual average sunshine time of 2,163.8 h, and annual average rainfall of 635.1–663.9 mm. One thousand leaves were collected from east, west, south and north branches in the middle part of 25 chosen trees. The samples were air-dried in the shade at room temperature, powdered and stored at −18 °C before extraction.

Rutin trihydrate, quercetin, chlorogenic acid, 1,1-diphenyl-2-picrylhydrazyl (DPPH), Folin-Ciocalteu’s phenol reagent,[4,4-[3-(2-pyridinyl-1,2,4-triazine-5,6-diyl) bisbenzenesulfonic acid] (ferrozine), and linoleic acid were purchased from Sigma-Aldrich Co. LLC. (Shanghai, China). Geniposidic acid, aucubin were purchased from National Institutes for Food and Drug Control (Beijing, China). All other chemicals were of analytical grade.

### 3.2. Preparation of Extracts

Each sample of the air-dried and ground leaf of EU (20 g, 20 mesh) was extracted twice with distilled water (200 mL) at 60 °C for 60 min. The two extracts were combined, filtered and evaporated to dryness under vacuum at 50 °C to give brown and yellow residues. The sealed extracts were stored at −18 °C before analysis.

### 3.3. Antioxidant Capacity Assays

#### 3.3.1. Antioxidant Activity in Linoleic Acid Emulsion

The antioxidant activity of EU leaf was determined according to the thiocyanate method [32]. The inhibition effect was calculated according to Equation (1):

Inhibition effect (%) = 100 − (absorbance of sample/absorbance of control) × 100 (1)

#### 3.3.2. DPPH Radical Scavenging Activity

The effect of EU leaf on DPPH radical was estimated according to a published method [33] with some modifications. A solution of samples in ethanol (2 mL) was added to 500 μmol/L DPPH^•^ solution (1 mL). After the mixture had been shaken, the reaction solution was allowed to stand for 20 min at room temperature in the dark. The absorbance of the mixture was measured at 517 nm. Solution (1 mL) of 500 μmol/L DPPH^•^ mixed with alcohol (2 mL) was used as the control. The radical scavenging activity of the samples was calculated according to Equation (1).

#### 3.3.3. Hydroxyl Radical Scavenging Assay

Hydroxyl radical scavenging activity was determined according to the method of Chung *et al*. [34]. The hydroxyl radical scavenging activity was calculated using Equation (1).

#### 3.3.4. Fe^2+^ Chelating Ability Determination

The Fe^2+^ chelating ability was determined according to a published method [5,35] with some modifications. A sample (1 mg/mL distilled water, 5 mL) was incubated with a solution of 2 mmol/L FeCl_2_ (0.1 mL) at room temperature for 10 min. The reaction was started by the addition of 5 mmol/L ferrozine solution (0.2 mL) and the reaction mixture was shaken and left to stand for 10 min at room temperature. After incubation, the absorbance of the solution was measured at 562 nm, and distilled water (5 mL) mixed with a solution of 2 mmol/L FeCl_2_ (0.1 mL) and 5 mmol/L ferrozine solution (0.2 mL) was used as a control. The lower absorbance of the reaction mixture indicated a higher Fe^2+^-chelating ability. The capability of chelating the ferrous ion was calculated using Equation (1).

#### 3.3.5. Antioxidant Activity in Rapeseed Oil

Extracts of EU leaf, or compounds (0.1 g) were added to rapeseed oil (50 g). All the samples were placed in dark brown colored reagent bottles with narrow necks, without stoppers and stored in an oven at a fixed temperature of 70 °C. Control samples were also placed under the same storage conditions. Analyses were carried out after 2 days (48 h). At least three samples of each category were analyzed. Peroxide Value (POV) measurement was made at regular intervals following an official method [36].

### 3.4. Determination of Contents of Antioxidants

#### 3.4.1. Total Phenolics

Total phenolics were estimated according to a protocol similar to that of Slinkard and Singleton [37]. One mL of sample solution was mixed with distilled water (5 mL) and 1 mol/L Folin-Ciocalteu’s phenol reagent (0.5 mL). The mixture was allowed to react for 5 min and 5 g/100 mL Na_2_CO_3_ (1 mL) was added. Thereafter, it was thoroughly mixed and placed in the dark for 1 hour and the absorbance was measured at 725 nm with the spectrophotometer. A gallic acid standard curve was obtained for the calculation of phenolics content. The amount of phenol was expressed as gallic acid equivalents (GAE) in milligrams per gram of dry plant material.

#### 3.4.2. Flavonoids

The flavonoids content of EU leaf was determined using a modified colorimetric method [38]. The flavonoids content was calculated by a rutin standard curve and expressed as rutin equivalents in milligrams per gram of dry plant sample.

#### 3.4.3. Chlorogenic Acid and Geniposidic Acid

High performance liquid chromatography (HPLC) was performed to determine the contents of chlorogenic acid and geniposidic acid in the samples [39]. A Shimadzu HPLC system (LC-10AT) equipped with a UV detector (SPD-10AVP), a high pressure pump, a Shim-pack VP-ODS column (150 mm × 4.6 mm, 5 µm), and a manual sample injector was used. The chromatographic conditions were as follows: mobile phase: ethanol–water–acetic acid (24:75:1, V/V); flow rate of the mobile phase: 1 mL/min; injection volume: 5 µL; detection wavelength: 240 nm; column temperature: 30 °C. The contents of chlorogenic acid and geneposidic acid were calculated based on the peak areas in the chromatogram.

#### 3.4.4. Rutin, Quercetin

A HPLC method was employed [18] to measure the contents of rutin and quercetin in the leaves which were collected in different time with the same HPLC system mentioned above. The chromatographic conditions were as follows: mobile phase: methanol–water–phosphoric acid (50:49.5:0.5, V/V); flow rate of the mobile phase: 1.0 mL/min; injection volume: 20 µL; detection wavelength: 270 nm; column temperature: 25 °C.

#### 3.4.5. Aucubin

An improved method was adopted by using dimethylaminobenzaldehyde as a colorimetric reagent to determine the content of aucubin [39].

### 3.5. Statistical Analysis

All results were obtained from three independent experiments and expressed as mean ± SD. Differences between treatments (*p* < 0.05) were determined by Duncan’s multiple range test.

## 4. Conclusions

After investigations of three years of samples of EU leaves, seasonal variation of some active compounds and antioxidant activity were proven. High content of phenolics was found in EU leaves collected in August, and the samples collected in May contained higher amount of flavonoids than other samples in the three years. May or June collection was related to high contents of rutin, quercetin, geniposidic acid and aucubin. Moreover, the August leaves showed strong DPPH radical scavenging activity, and metal ion chelating ability. May samples showed a potential to become a food antioxidant because of its strong inhibition effects, as potent as BHT, on oxidation of rapeseed oil and linoleic acid in the three years. Correlations between the antioxidant activities and the contents of active compounds were found. The DPPH radical scavenging activity was related to total phenolics content more closely than that of flavonoids. However, flavonoids played an important role in the inhibition effects on rapeseed oil and linoleic acid oxidation. Therefore, August and May are recommended to be an appropriate time to harvest leaves of *Eucommia ulmoides* Oliver for industry.

## References

[1] Nakasa T., Yamaguchi M., Okinaka O., Metori K., Takahashi S. (1995). Effects of Du-zhong leaf extract on plasma and hepatic lipids in rats fed on a high-fat plus high cholesterol diet. Nippon Nogeikagaku Kaishi.

[2] Nakazawa Y. (1997). Functional and healthy properties of Du-zhong tea and their utilization. Food Ind..

[3] Nakamura T., Nakazawa Y., Onizuka S., Satoh S., Chiba A., Sekihashi K., Miura A., Yasugahira N., Sasaki Y.F. (1997). Antimutagenicity of Tochu tea (an aqueous extract of *Eucommia ulmoides* leaves). 1. The clastogen-suppressing effects of Tochu tea in CHO cells and mice. Mutat. Res..

[4] Sasaki Y.F., Chiba A., Murakami M., Sekihashi K., Tanaka M., Takahoko M., Moribayashi S., Kudou C., Hara Y., Nakazawa Y. (1996). Antimutagenicity of Tochu tea (an aqueous extract of *Eucommia ulmoides* leaves). 2. Suppressing effect of Tochu tea on the urine mutagenicity after ingestion of raw fish and cooked beef. Mutat. Res..

[5] Hsieh C.L., Yen G.C. (2000). Antioxidant actions of Du-zhong (*Eucommia ulmoides* Oliv.) toward oxidative damage in biomolecules. Life Sci..

[6] Yen G.C., Hsieh C.L. (2000). Reactive oxygen species scavenging activity of Du-zhong (*Eucommia ulmoides* Oliv.) and its active compounds. J. Agric. Food Chem..

[7] Yen G.C., Hsieh C.L. (1998). Antioxidant activity of extracts from Du-zhong (*Eucommia ulmoides Oliv*) toward various lipid peroxidation models *in vitro*. J. Agric. Food Chem..

[8] Zhang Q., Su Y.Q., Yang F.X., Peng J.N., Li X.H., Sun R.C. (2007). Antioxidative activity of water extracts from leaf, male flower, raw cortex and fruit of *Eucommia ulmoides* Oliv. For. Prod. J..

[9] Young H.S., Park J.C., Park H.J., Lee J.H., Choi J.S. (1991). Phenolic compounds of the leaves of *Eucommia ulmoides*. Arch. Pharm. Res..

[10] Cheng J., Zhao Y., Cui Y., Cheng T. (2000). Studies on flavonoids from leave of *Eucommia ulmoides* Oliv. Zhong Guo Zhong Yao Za Zhi.

[11] Takamura C., Hirata T., Ueda T., Ono M., Miyashita H., Ikeda T., Nohara T. (2007). Iridoids from the green leaves of *Eucommia ulmoides*. J. Nat. Prod..

[12] Chen Z., He J., Tang D., Shi Q., Li Q. (2004). Nutrient distribution and uptake characteristics in Fucommia ulmoides Oliv. plant. J. Northwest For. Coll..

[13] Deyama T., Nishibe S., Nakazawa Y. (2001). Constituents and pharmacological effects of Eucommia and Siberian ginseng. Acta Pharm. Sin..

[14] Rice-Evans C. (2001). Flavonoid antioxidants. Curr. Med. Chem..

[15] Ma H.L., Han X.W., Dong J.E., Zhang B.Y., Zhang A.L. (2003). Study on the relation between the dynamic accumulation of secondary metabolites and the phenological period of *Eucommia ulmoides* Oliv. Acta Botanica Boreali Occidentalia Sinica.

[16] Qi X.Y., Chen W.J., Zhang S.H. (2004). Studies on distribution and accumulative dynamic variation of bioactive components in *Eucommia ulmoides*. Chin. Trad. Herbal Drug.

[17] Zhang A.L., Ma Y.T., Zhao D.Y., Gao J.M., Zhang K.J. (2009). Study on effects of seasonal and regional differences on secondary metabolites of *Eucommia ulmoides* Oliv. leaves. Chem. Ind. For. Prod..

[18] Chen L., Yang Z. (2007). Study on dynamic variation of three flavonoids contents in folium eucommiae harvested from different seasons. Strait Pharm. J..

[19] Zhang K.J, Ma X.H., Ma M., Wang L., Zhang T. (1999). A study on dynamic accumulation of metabolites during the growth of *Eucommia ulmoides* Oliv. Scientia Silvae Sinicae.

[20] Aruoma O.I. (1994). Nutrition and health aspects of free radicals and antioxidants. Food Chem. Toxicol..

[21] Clifford M.N. (2000). Chlorogenic acids and other cinnamates—Nature, occurrence, dietary burden, absorption and metabolism. J. Sci. Food Agric..

[22] Heim K.E., Tagliaferro A.R., Bobilya D.J. (2002). Flavonoid antioxidants: Chemistry, metabolism and structure-activity relationships. J. Nutr. Biochem..

[23] Frankel E.N., Meyer A.S. (2000). The problems of using one-dimensional methods to evaluate multifunctional food and biological antioxidants. J. Sci. Food Agric..

[24] Cheeseman K., Slater T. (1993). An introduction to free radical biochemistry. Br. Med. Bull..

[25] Manzocco L., Anese M., Nicoli M.C. (1998). Antioxidant properties of tea extracts as affected by processing. Food Sci. Technol..

[26] Gulcin I., Sat I.G., Beydemir S., Elmastas M., Kufrevioglu O.I. (2004). Comparison of antioxidant activity of clove (*Eugenia caryophylata* Thunb) buds and lavender (*Lavandula stoechas* L.). Food Chem..

[27] Sanchez-Moreno C. (2002). Review: Methods used to evaluate the free radical scavenging activity in foods and biological systems. Food Sci. Technol. Int..

[28] Diplock A.T. (1997). Will the “Good fairies” please prove to us that vitamin E lessens human degenerative disease?. Free Radic. Res..

[29] Yildirim A., Mavi A., Kara A.A. (2001). Determination of antioxidant and antimicrobial activities of *Rumex crispus* L. extracts. J. Agric. Food Chem..

[30] Hsu C.L., Chen W.L., Weng Y.M., Tseng C.Y. (2003). Chemical composition, physical properties, and antioxidant activities of yam flours as affected by different drying methods. Food Chem..

[31] Robards K. (2003). Strategies for the determination of bioactive phenols in plants, fruit and vegetables. J. Chromatogr. A.

[32] Mitsuda H., Yuasumoto K., Iwami K. (1996). Antioxidation action of indole compounds during the autoxidation of linoleic acid. Eiyo to Shokuryo.

[33] Tanaka N., Nishikawa K., Ishimaru K. (2003). Antioxidative capacity of extracts and constituents in Cornus capitata adventitious roots. J. Agric. Food Chem..

[34] Chung S.K., Osawa T., Kawakishi S. (1997). Hydroxyl radical-scavenging effects of spices and scavengers from brown mustard (*Brassica nigra*). Biosci. Biotechnol. Biochem..

[35] Decker E.A., Welch B. (1990). Role of ferritin as a lipid oxidation catalyst in muscle food. J. Agric. Food Chem..

[36] Zhang L., Yang X., Zhang Y., Wang L., Zhang R. (2011). *In vitro* antioxidant properties of different parts of pomegranate flowers. Food Bioprod. Process..

[37] Slinkard K., Singleton V.L. (1977). Total phenol analysis: Automation and comparison with manual methods. Am. J. Enol. Vitic..

[38] Jia Z.S., Tang M.C., Wu J.M. (1999). The determination of flavonoid contents in mulberry and their scavenging effects on superoxide radicals. Food Chem..

[39] Dong J.E., Ma X.H., Wei Q., Peng S.B., Zhang S.C. (2011). Effects of growing location on the contents of secondary metabolites in the leaves of four selected superior clones of *Eucommia ulmoides*. Ind. Crops Prod..

